# In a demanding task, three-handed manipulation is preferred to two-handed manipulation

**DOI:** 10.1038/srep21758

**Published:** 2016-02-25

**Authors:** Elahe Abdi, Etienne Burdet, Mohamed Bouri, Sharifa Himidan, Hannes Bleuler

**Affiliations:** 1 Robotic Systems Laboratory, École Polytechnique Fédérale de Lausanne (EPFL), Lausanne, Switzerland; 2 Department of Bioengineering, Imperial College of Science, Technology and Medicine, London, United Kingdom; 3 Pediatric General and Thoracic Surgery, Hospital for Sick Children, University of Toronto, Toronto, Ontario, Canada

## Abstract

Equipped with a third hand under their direct control, surgeons may be able to perform certain surgical interventions alone; this would reduce the need for a human assistant and related coordination difficulties. However, does human performance improve with three hands compared to two hands? To evaluate this possibility, we carried out a behavioural study on the performance of naive adults catching objects with three virtual hands controlled by their two hands and right foot. The subjects could successfully control the virtual hands in a few trials. With this control strategy, the workspace of the hands was inversely correlated with the task velocity. The comparison of performance between the three and two hands control revealed no significant difference of success in catching falling objects and in average effort during the tasks. Subjects preferred the three handed control strategy, found it easier, with less physical and mental burden. Although the coordination of the foot with the natural hands increased trial after trial, about two minutes of practice was not sufficient to develop a sense of ownership towards the third arm.

Many tasks in open surgery, laparoscopic and micro- surgery need one or several assistants e.g. to hold a suction tube to clear blood, direct the camera in laparoscopic surgery and push organs aside. In microsurgery, delicate tissues should be held during long intervals in a fixed position for suturing, which is difficult for humans. If the assistant holding the camera is novice or unfamiliar with the surgeon, the assistant can often have problems in positioning it appropriately in the 3D space, may be confused with the fulcrum effect and suffer from fatigue^1^. In laparoscopic surgery, some types of suture require three instruments in addition to the camera. The third instrument is operated by an assistant to hold the thread and improve knot stability. We envision that a robotic arm under the surgeon’s control could enable him or her to carry out many tasks alone. This could for example facilitate the suturing process as all three instruments would be controlled by the same brain, which may ensure more coherent movements than by collaborating with an assistant. In addition, this will reduce the physical burden on the assistants involved in tasks demanding high precision, high force and/or minimum possible tremor. Finally solo surgery operations are less costly^2^. Surgical team members have to maintain sterile hands and gloves. Apart from direct operational assistance, an unsterile arm may be useful for the surgical team to touch and manipulate unsterile objects in the operating room without infecting their hands e.g. adjusting the parameters of a device through pushing its buttons. Also in emergency situations, with reduced availability of surgical staff, a surgeon with a robotic arm under his or her control may be capable of performing a surgery with fewer human assistants.

The idea of having a robotic arm helping humans in the operating room is not new. Camera holders are among the most common robotic surgical assistant devices. They are either motion controlled (e.g. EndoAssist by head motion tracking^3^,^4^,^5^), voice activated (e.g. AESOP^6^ and Image Tracking System^7^), or simply commanded by a joystick (e.g. LapMan^8^). It has been shown that robotic camera holders can replace the human camera holder and may be more convenient to the surgeon^7^. Positioning of passive camera holders, such as TISKA developed in 1999, by the surgeon who adjusts it using one hand and locked or unlocked by a foot switch^9^, have been reported as more time consuming but more comfortable compared to human assistants^10^. Providing the surgeon with direct control on the cameras position in laparoscopic surgery improves the surgeon’s ability to perform explorative and manipulative tasks^11^. In AESOP, the foot control is faster with less operator-interface failure, but the voice control is more accurate^12^. However, subjects learned to work with the foot control faster than with voice control.

A good control of a robotic assistant will cause minimal distraction to the user. Ideally, the robotic arm should be perceived by the surgeon as a supernumerary limb that can be commanded as intuitively as the natural limbs. The first evidence that the perceptual illusion of owning an artificial hand could be induced, was provided by the rubber hand illusion in 1998^13^. New studies about the rubber hand illusion show that congruent mapping between the real and artificial hands is necessary for developing sense of ownership towards the rubber hand^14^, and the morphological similarities between the tool and the body part favours the induced embodiment^15^. Even a mechanical hand made of wires can result in a sense of embodiment, although the effect is not as strong as that of a realistic rubber hand^16^. The literature suggests that parallel to development of sense of ownership towards the rubber hand, the sense of ownership towards the real hand decreases^17^. Other studies show that while multiple supernumerary limbs can be incorporated into the bodily image (i.e., the sense of ownership towards the supernumerary limb), only one can be included in the body schema (i.e., the ability to control the supernumerary limb)^18^,^19^. Bodily perception can be experimentally modified through visual cues, functional adaptation and embodiment of tools and prostheses^20^,^21^. Research proves the plasticity of the human brain not only to master the use of external tools but also to reshape the body representation^22^.

Previous studies have proven the feasibility of solo surgery although new strategies are required for better control of the robotic assistive devices and for increasing their precision^23^. We propose the use of the foot and leg for controlling a third robotic arm not only for positioning a camera in laparoscopic surgery but also for doing other simple tasks such as retracting an organ or holding the thread in suturing^24^. Evidence from car driving, playing musical instruments such as percussion, etc. suggest that it is possible to use the foot for relatively complex tasks and with little distraction. We note that, although our final goal is to develop a third robotic arm application for surgical tasks, the results of the present study may be used in other fields that can potentially benefit from additional limbs. Supernumerary arms may be useful in industrial applications e.g. holding a heavy drill to decrease the physical burden on the worker^25^. Also supernumerary fingers can enhance the functionality of the hand in tasks that are usually too difficult to be carried out by one hand e.g. grasping a large object or taking the lid off a jar^26^.

The state of the art in cognitive neuroscience research suggests that it is possible to develop a sense of ownership towards a supernumerary limb. However, it is not at all clear whether using three hands (two biological hands plus a robotic arm) improves the performance compared to using two hands in a demanding task. It is also important to assess the users’ preference for different possible control strategies with two or three hands. The present study aims at investigating such questions. An experiment is designed in virtual reality in which the same task is performed with two virtual hands vs. with three virtual hands. The third hand is controlled by tracking foot movements. The functional differences in limb usage in two handed and three handed scenarios are investigated. The performance of the subjects and the physical and mental burden of the tasks are compared. The learning curves of the participants, their sense of ownership towards the third hand and the ease of control of the third hand are analysed.

## Results

The experiment consists of catching three virtual falling polygons once using two hands (2 h) that mimic the movements of the two real hands of the user, and another time using three hands (3 h); the third hand is controlled by the foot. In the 2 h scenario, the left hand can catch the left and middle objects while the right hand is able to catch the right and middle objects. In the 3 h scenario, left and right hands can only catch the left and right objects respectively while the middle object can only be caught by the foot controlled virtual hand. The target zone is a circle centred at the centre of the falling object and with a diameter equal to the polygon’s smallest edge. Left and right hands can catch an object by pinching inside the target zone, however, the foot-controlled hand should only get inside the zone to catch. Each experiment involves three game rounds. The objects’ falling speed is doubled in the second and third rounds compared to their respective previous round. The experimental setup and paradigm are explained in detail in the Methods section.

The results are presented for the average performance of all the participants as well as left- and right-handed subjects. No significant difference between females’ and males’ performance was detected in any of the measures analysed below, thus we do not differentiate the subjects according to their sex in the following. Subjects’ performance is assessed through objective measures of efficiency, workspace, smoothness, velocity, limb’s simultaneous action, number of pinches by each hand and effort. Table 1 presents a summary of average objective performance measures over all the participants in the total time spent in each of the 2 h and 3 h scenarios. Each measure will be presented in detail in this section.

### Efficiency

26 of the 35 subjects managed to finish the game with two hands and 25 subjects managed to finish the three hands game 3 h. The 2 h game was finished in 127 ± 7 s and the three handed one in 126 ± 9 s. Slightly fewer objects were lost in the 3 h scenario (3.8 ± 0.8 for the 2 h game vs. 3.5 ± 0.8 for 3 h). There are large individual performance differences among the subjects. In 2 h there was a direct relation between the increase in game’s speed and the number of lost objects i.e. as the speed increased more objects were lost. However, there was no such relation in 3 h. In this paradigm, the performance got worse in the second game round compared to the first one but then improved in the third round. In the maximum speed condition, 46% less objects were lost in 3 h compared to 2 h.

### Workspace

**Figure 1:**
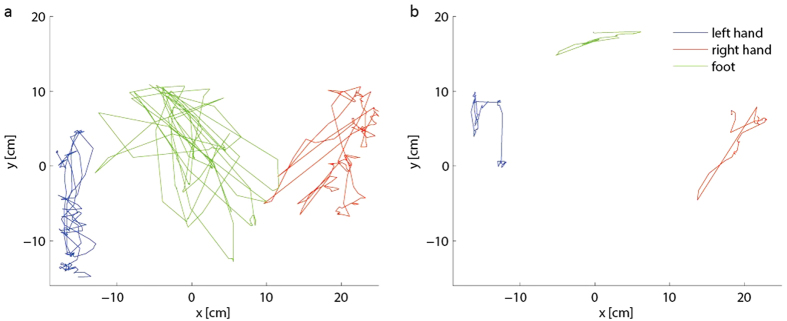
The second game round. (**a**) Some subjects moved their limbs within a rectangular workspace. (**b**) Some subjects moved their limbs in a linear manner.

It is useful, in the design of control interfaces, to know the comfortable workspace of the limbs with respect to each other and as a function of movement velocity. Right-handed participants used the right hand for catching the middle object in 63 ± 6% of the cases and left-handed participants used the left hand in 69 ± 11% of the cases. In the three-handed game, different subjects had quite different strategies in moving their limbs. Some moved their limbs in a rectangular space, whereas others performed actions with minimal movements resulting in nearly linear workspaces (Fig. 1).

**Figure 2:**
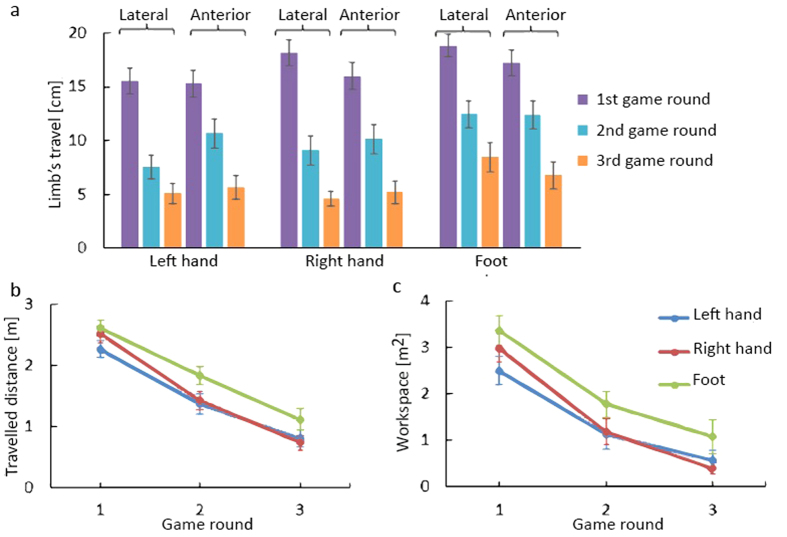
(**a**) Limbs’ travel distance in- Top: lateral and anterior directions, (**b**) Cumulative travelled distance of left hand, right hand and foot in the three game rounds of the three-handed game, (**c**) Workspaces of left hand, right hand and foot in the three game rounds of the three handed game.

The distance travelled decreased as game speed increased, both in the lateral and anterior directions for every limb (Fig. 2a). The *workspace* of each limb is defined as the smallest rectangle containing all the paths of that limb. The size of the workspace was negatively correlated with the falling speed of the objects such that the workspace decreased of half when the velocity was doubled in each game round (Fig. 2b). Figure 2c shows the average cumulative distance travelled over subjects for each limb and each game round. The distance the hands travelled decreases linearly through the game rounds, but for the foot the distance travelled remains almost constant from the second to the third game round.

### Smoothness

**Figure 3:**
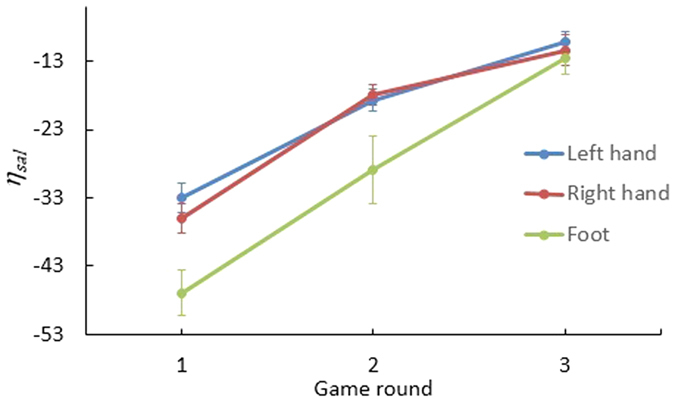
Smoothness of the movements of each limb using the spectral arc length metric.

The smoothness of the movements of each limb was quantified using the spectral arc length metric (η_sal_)^27^. It was independent of the sex and dominant hand of the subjects. Figure 3 presents the average smoothness of the limbs’ movements over the subjects in the three game rounds. On average over all subjects, the smoothness improved 45% in the second and third game rounds with respect to the previous round. In the first round, the foot was more jerky, however it improved in subsequent rounds with a value similar to the hand in only three rounds (left hand: −10.25, right hand: −11.40, foot: −12.49).

### Velocity

**Figure 4:**
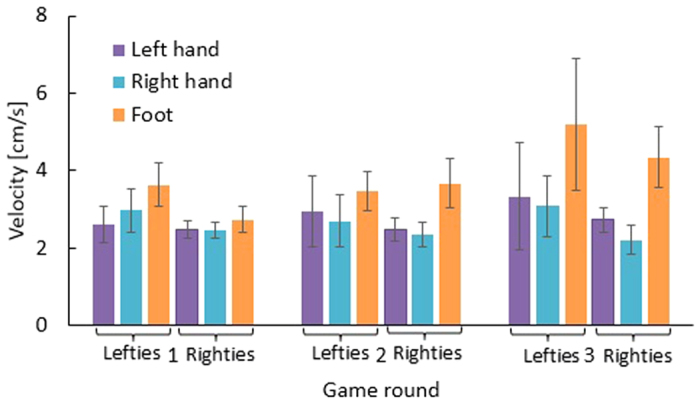
Left hand, right hand and foot velocities for right handed and left handed people in different game rounds.

Subjects were free to use each of their hands to catch the middle object in 2 h, consequently the hands’ speed depends on the hand’s choice. However, in 3 h subjects are constrained to use each of their three limbs (two hands and one foot) for catching a specific object. The results of Fig. 4 show that the difference between limbs’ velocities became larger as the game speed increased in consecutive game rounds for both left and right-handed subjects. Over all subjects, the average velocities of two hands were not noticeably different (right hand: 2.9 ± 0.7 cm/s, left hand: 3 ± 0.9 cm/s). The foot was faster (4.2 ± 0.9 cm/s) but the velocity difference among the limbs was not significant.

### Limbs’ action

**Figure 5:**
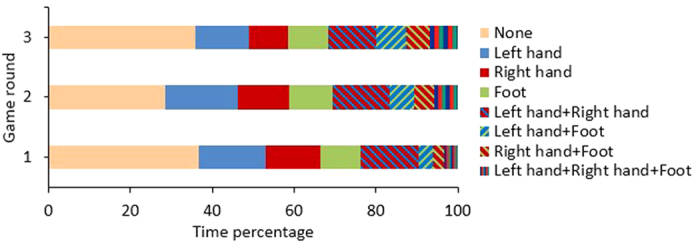
Average individual and simultaneous action of the three limbs in three consecutive game rounds.

In the two-handed game, the *simultaneous action* (defined as the time during which both hands are moving simultaneously) increased constantly through game rounds from 23% to 44% of the total time of the game. In the three-handed experiment there was less simultaneous movement between one of the hands and the leg than between the two hands (Fig. 5). Also, on average for all subjects, in 34% of the total time none of the limbs were moving.

The mean time percentage of periods of simultaneous movements during the whole game and over the participants for each combination of two limbs was: right hand-left hand: 18.5%, left hand-foot: 11%, right hand-foot: 9.7%, both hands and the foot: 5.3%. Males and females had almost the same rate of simultaneous movement between the limbs with a maximum difference of 2.8% in any combination. A remarkable characteristic is that over the game rounds the proportion of simultaneous movements for foot and either hand or with the two hands increased monotonically. On average over all the subjects, this increase was 74% from the first to the second round, which is a significant difference (p < 0.02, z = −2.4197, rank sum test) and 19% from the second to the third round, which is not significant.

The number of pinches for catching objects decreased with increasing speed. Left-handed subjects pinched significantly more with their left hand (p < 0.03, z = 2.25, rank sum test) compared to right-handed subjects. Both groups pinched more with their dominant hand compared to the non-dominant hand. 52 ± 5% of pinches in the two handed scenario and 58 ± 6% of them in the three handed one are done in the target area i.e. over the object.

**Figure 6:**
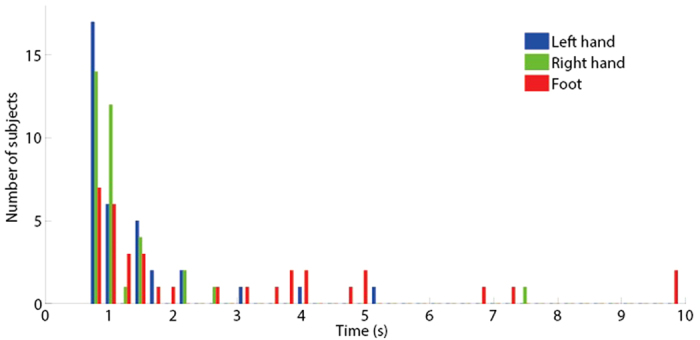
Histogram of the instance of action initiation of each limb across the participants.

The histogram of the instance of action initiation of each limb shows that all the limbs moved within 10 seconds from the start of the game (Fig. 6). In most cases the foot was the last limb that moved. However, there is no systematic order in the limbs’ movements, i.e., the different limbs move in parallel rather than serially.

### Effort

We define effort as the work per mass. This measure gives insight into the amount of consumed energy in each of the two handed and three handed experimental strategies and it can be used as a metric of performance efficiency. Work is defined in Eq.1. In this equation, the parameters are defined as follows: *W*: work, *F*: force, m: mass, *s*: displacement, 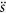: acceleration, *t*_*0*_: the time at which the game starts, *t*_*e*_: the time at which the game ends.


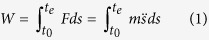


By discretising Eq.1, work per mass (effort) can be derived as a function of displacement (*Δs*_*i*_) in each time step *(Δt* = 0.2 s) as presented in Eq.2. The summation is over the sampling period starting at *t*_*0*_ (*i* = 1) and ending at *t*_*e*_ (*i* = n).


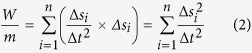


**Figure 7:**
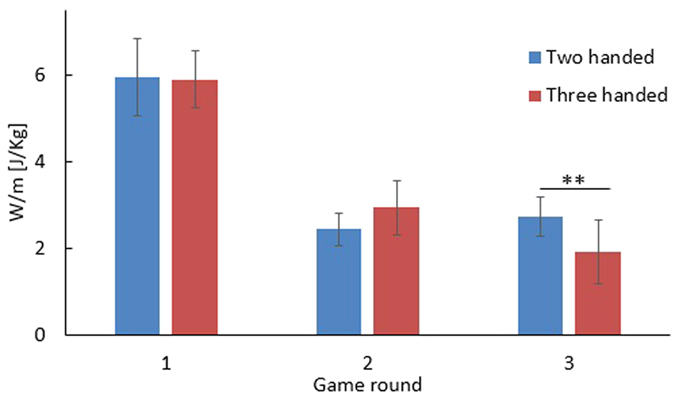
Work performed in each game round in the two handed and three handed scenarios.

The average work per mass of the two and three handed scenarios were similar, with a small difference of 3%. Fig. 7 presents the work performed in each game round and control strategy. In the second game round, the effort in the three handed scenario was 16% larger than that of the two handed scenario but the difference was not significant. In the third and faster game round the work per mass of the three handed scenario was 30% less than that of the two handed one which is a significant difference (p < 0.001, z = 3.4381, rank sum test).

### Subjective assessment

**Figure 8:**
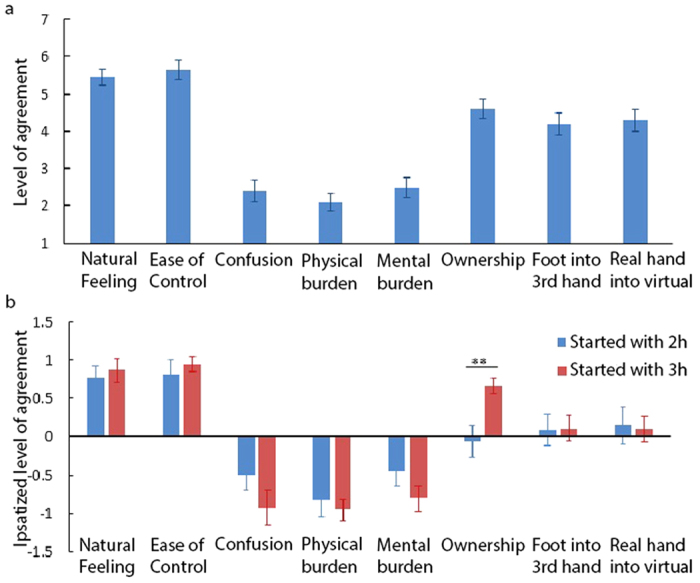
(**a**) Average response of all the participants to the questionnaire of Table 1. 1: Strong disagreement, 7: Strong agreement (SEM is represented), (**b**) Ipsatized mean questionnaire ratings for those who started with 2 h and those who started with 3 h.

Figure 8a illustrates the results of the questionnaire about the three handed control strategy over all the subjects. The differences between the responses of those who started the experiment with the two handed game and those who started with the three handed one are reported along with the global results (Fig. 8b). To obtain a more accurate comparison between the two groups, the data from each question were standardised using an ipsatization procedure. Ipsatized data account for uniform response biases^28^, i.e. participants’ answers will be ranked according to their personal understanding of the rating scale. It was calculated by subtracting the mean rating of all the responses of a subject from each of his or her responses and dividing it by the standard deviation of the subject’s responses in all the questions. It is indicated in the text wherever the ipsatized data is reported.

Subjects found it natural to control three hands simultaneously (Q1, mean response: 5.5 on the range [1,7] with 4 as mid-range value). Those who started with the three handed game found it more natural to control the third hand by foot compared to those who started from the two handed one but the difference is not significant. It has also been easy for the subjects to control the third hand by foot (Q2, mean response: 5.7). Those who started with the three handed game found it slightly easier. Participants were not confused by the number of tasks they had to perform at the same time with three virtual hands (Q3, mean response: 2.4, where 1 = least confusion). Those who started with the three handed game have reported slightly less confusion (not significant), indicating that doing the same task with two hands did not result in participants feeling more comfortable in the three handed strategy. Physical and mental burdens are low (under 2.5) for the whole sample (Q4 and Q5).

Participants were almost neutral about having sense of ownership towards the third hand (Q6, mean response: 4.6). However, those who started with the three handed experiment had an average ipsatized response of 0.66 which is significantly higher (p < 0.004, t = −3.1765, t-test) compared to those who started with the two handed game with an average ipsatized response of −0.06. Not only do these results indicate that performing a task with two hands before the three handed experiment does not help the participant feel more comfortable in the three handed game, but it also produces an unwanted reference which negatively influences the user’s perception of the three handed task.

Subjects did not feel that their foot was turning into the third arm (Q7). Mean answer for all the participants to the corresponding question is 4.2, indicating that the sense of ownership towards the third hand is not as a replacement of an existing limb (here the foot) but it is perceived as a virtual supernumerary limb. Answers to the control question were independent of the succession of the games and it was almost neutral (Q8, mean response: 4.3), illustrating that subjects did not feel that their real hands were turning into virtual limbs. We expected negative or neutral answers to this question indicating that subjects have not answered the questions in a random manner.

**Figure 9:**
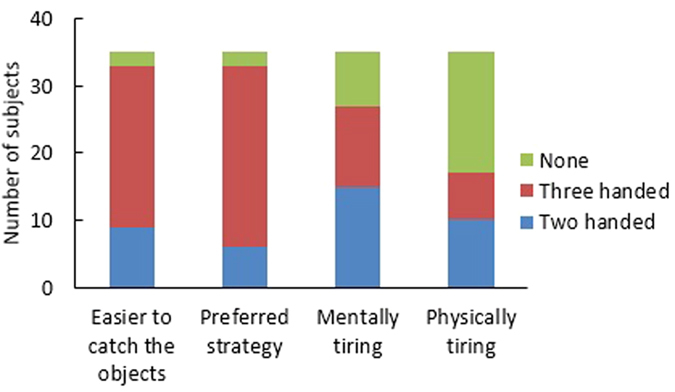
Average response of all participants to the comparative questionnaire.

The comparative questionnaire revealed that the three-handed game was better accepted in every sense (Fig. 9). 69% of the participants found the three handed game easier for catching three objects and 77% preferred the three handed strategy for this game indicating that if a task demands more than two hands, subjects are willing to use more hands to perform the task. 29% of subjects found the two handed game physically more tiring, 20% reported that the three handed game was more tiring and 51% thought none of the games were tiring. 43% of participants thought the two handed game was mentally tiring, 34% thought three handed game was mentally tiring and 23% said that none of the games were mentally tiring. The responses to the last two questions suggest that the added effort for catching the middle object with one of the two hands is mentally and physically more demanding. The smaller physical and mental burden for the three-handed game also explains why it is preferred and found easier.

## Discussion

An experimental study was conducted to analyse and compare humans’ performance in doing a demanding task with two or with three hands. The experiment was carried out in virtual reality where the third hand was controlled by the right foot. The virtual hands were controlled by motion of the real hands and the foot tracked by two Kinect cameras^29^.

Previous studies have typically investigated the factors influencing the bimanual or one hand and one foot coordination^30^,^31^. To our knowledge, the present study is the first on bimanual versus three handed manipulation performance in which the two hands and one foot perform similar tasks simultaneously and independently.

The two hands moved at similar velocities on average, while the foot always moved faster than the hands. This shows that the foot interface should compensate for the faster and more jerky foot movements in order to control a robotic arm with the same speed characteristics as biological arms. This ultimately may help coordination of the three arms. The three limbs were moved simultaneously in less than 10% of the time through the game rounds. This shows that performing three independent tasks with three limbs is not achievable within a few minutes of practice and it is not the best approach to multi-limb applications. The two hands had more simultaneous movement compared to one hand and one foot. Ipsilateral hand and foot had the least simultaneous movement among all the possible combinations of the limbs. It is known that ipsilateral limbs have the most inaccurate simultaneous movements in opposite directions^32^. We believe this is the reason they were voluntarily less likely to be chosen for simultaneous actions in our experiment. Interestingly, the time in which the foot was used together with one or the two hands increased uniformly over the game rounds. Although the task in this experiment can be completed by sequential movement of the limbs, more objects could be caught by simultaneous usage of limbs. The increase in simultaneous movements through the game rounds proves that subjects adopted a more efficient strategy within a few minutes of practice. Limbs started their movement simultaneously i.e. there was no preference in the sequence of moving the limbs for the first time in the game. A previous study on dual, driving like, simultaneous and independent task of the two hands and one foot has shown that attention to concurrent reaching with hands and foot pedal tracking is flexibly allocated based on task structure and priority^33^. The present study shows that proposing carrying out the same task for the limbs results in a parallel non-prioritised control of the limbs. The working space as well as the distance travelled by each limb were inversely proportional to the movement velocity in each game round. It shows that the more demanding the task, the more attention is devoted to performance efficiency i.e. minimizing the path between the hand and the target object. The participants used different performance strategies; some of them actively moved their limbs through the game, while others tended to optimize their performance by minimizing their movements. The individual differences are also illustrated in the subjects’ efficiency e.g. the number of caught objects, the time required to complete the game and the pinching accuracy. As a result, a global instructional program for using the third hand should be backed up with a study on the performance of a large sample to make it suitable for every user.

On average over all the participants and conditions, there was no significant performance difference between the two control strategies. However, at maximum game speed, objects were lost twice as frequently in the two-handed as in the three handed game. This shows that the three-handed control is more effective once the task becomes more demanding. A previous study on hands-foot coordination in gaming, reports less cognitive burden in independent tasks^34^, however they proposed different tasks for the hands and the foot. Subjective assessment in the current study, which consists of independent but similar tasks for all the limbs, shows that participants found the three handed control strategy natural and were not confused by the paradigm. They found it easier to play the game with three hands and preferred this strategy, reporting less physical and mental burden. The preference of the control paradigm did not depend on the effort used in each strategy, as the effort was almost the same for both.

Two minutes of practice was not enough for developing the sense of ownership towards the third hand. In our previous experiment with three different virtual games^35^, we observed that the sense of ownership towards the third hand improved constantly through the games. Also, performing different types of three handed tasks may enhance the ownership. Comparing the performance of the participants in that experiment and the one presented in this paper, we can see that in the previous experiment only an average of 1.5 objects were missed in each game whereas in the current experiment an average of 3.5 objects were missed. The average required time for completing the same game in the previous experiment was 9% less compared to the current experiment. These results highlight the effect of practice. The performance of participants improved significantly once they received a few minutes of practice with other games which needed three hands before they actually played the falling objects game.

In the current experiment, starting with the two handed game did not improve performance significantly relative to subjects who started directly with the three handed game. This indicates that performing the same task with two hands does not help in mastering the three hands paradigm.

The main limitations of this experiment are the sample group and the task type. Most of the subjects were students, thus younger than a typical surgeon and with an engineering background. Also the task is not comparable to a surgical manipulation. Therefore the present results cannot be applied directly to a surgical situation. Further testing will require involving surgeons and tasks closer to surgical gestures. However, it provides strong evidence of the usefulness of a third arm in demanding tasks from surgical to industrial application.

These preliminary results encourage us to address step by step the levels of growing complexity of manipulation. This might include embodiment of a third hand for positioning only (e.g. an endoscope, or holding an organ out of the way of the operation site), grasping with the third arm (e.g. a two fingered hand acting as a tweezer) and cooperation among different limbs. Each of these general steps has to be divided into more detailed scenarios with respective increased complexity.

## Conclusion

Hands-foot collaboration is widely used in tasks such as driving (vehicle control), playing certain musical instruments and more recently in gaming. However there is little published literature on the possibility of using a foot controlled supernumerary hand along with the two real hands for complex manipulation. This paper provided a comparison of performance during the same task carried out with two and three hands. The results show that in the selected three objects reaching task, subjects preferred to use three hands. They found it easier to complete the task with three hands and reported lower mental and physical burdens than when two hands were used. In fact, from the objective measure of caught objects, we learned that in the most demanding task (fastest game speed), the participants performed better with three hands. This suggests that a third arm can improve performance in applications that involve handling multiple tasks in a short period of time.

This study casts light on the users’ approach to the three handed control as compared to the two handed one. The findings suggest a high potential in using the foot to become more autonomous in surgery as well as other fields.

## Methods

### Experiment

**Figure 10:**
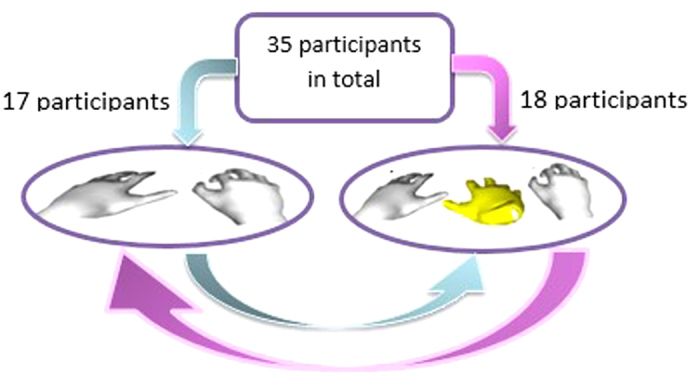
Experimental design: 35 subjects participated in the experiment. 17 of them stared with the 2 h and then performed the 3 h. 18 of them started with the 3 h and then performed the 2 h.

Thirty-five subjects with mean age 23 ± 5 years participated in the experiment. Thirteen were female and ten left-handed. The experiment was approved by the BMI Ethics Committee for Human Behavioural Research at EPFL and the methods were carried out in accordance with the approved guidelines. Informed consent was obtained from all subjects. The experiment was developed to investigate and compare the performance of participants in carrying out a demanding object grasping task using two distinct strategies: with two virtual hands (2 h) or with three virtual hands (3 h). All the subjects participated in both scenarios. 17 subjects started the grasping game with the 2 h strategy and then proceeded to the 3 h while 18 other participants started with 3 h and then proceeded to the 2 h, so that the influence of practice with one strategy or the other could be investigated (Fig. 10).

### Setup

The experiment is designed as a virtual game played with two or three virtual hands. Two virtual hands move on a computer monitor according to the movements of the two real hands of the player, while the third virtual hand is controlled by the player’s right foot, i.e. the third hand trajectory on the monitor corresponds to the foot’s planar movement on the floor. Two Microsoft XBOX 360 Kinect© depth cameras are used, one for tracking the movements of the player’s two hands and the other one for the foot. The software development kit (SDK) of the 3Gear Systems Company, which includes a library of predefined hand gestures, has been used to track the finger motions of the real hands. Each SDK supports only one camera, consequently a network of two PCs have to operate in parallel to render three virtual hands in real-time.

### Paradigm

**Figure 11:**
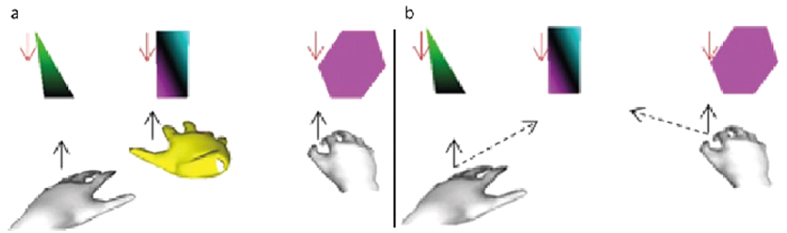
Catch the three falling objects. (**a**) Three handed strategy, each hand catches its corresponding object. The yellow virtual hand is controlled by the right foot. (**b**) Two handed strategy. The middle object can be caught by the left or the right hand.

A game has been implemented for the 2 h and 3 h-scenarios in which three polygons with different shapes falling from top to the bottom have to be caught before they reach the ground (Fig. 11). The left and right objects can be caught by pinching with the corresponding (grey) hands while the middle object has to be *touched* with the foot controlled (yellow) virtual hand. An object is “touched” when the centre of gravity of the (yellow) virtual hand gets within a circular target zone of the centre of the falling rectangle, where the diameter of the target zone corresponds approximately to the width of the rectangle.

When an object is caught, it disappears and a new object starts falling from top. The screen is divided into three columns of equal width. Each object stays in its allocated column but it doesn’t always fall from the same spot within its zone. This change in horizontal location forces the player to stay concentrated and active during the game. Each game has three rounds. In the first two rounds, if three samples of each object are caught, the next round starts with double falling speed compared to the previous round. In the last (fastest) round, if three samples of each object are caught, a win message will appear on the screen. In each round, if more than three samples from the same object are lost, the game will be over and a failure message appears on the screen.

In the 2 h paradigm, the left and right objects can be caught only with the respective hand while the middle object can be caught with either hand. The 3 h paradigm uses the translational movement of the right foot to control the third virtual hand. Three virtual hands appear on the screen that can catch three falling objects. The left and right objects are caught as in the 2 h experiment while the middle object can now only be caught by the third – foot controlled – virtual hand. In both scenarios, the user should pinch to catch the triangle and the hexagon. The rectangle object placed in the middle is “caught” when the virtual hand’s palm comes over it (with no need of any special gesture).

### Assessment

In each game, the planar position of the virtual hands are recorded at a frequency of 5 Hz and the hands’ movement velocity is then calculated. In addition, the time of pinches with the left and right hands are recorded. This is completed by a subjective assessment at the end of the three handed experiment through a questionnaire about different aspects of the control strategy, the sense of ownership towards the third hand, the perceived level of complexity of the task and the physical and mental burden of the game as described in Table 2. Each statement should be ranked in an ordered response Likert scale, from 1 (for “strong disagreement”) to 7 (for “strong agreement”). Question 8 is a control question.

At the end of the whole experiment, subjects answer four questions on the two different games (Table 3). These questions compare the two games with respect to the ease of the games, the control strategies as well as their mental and physical burden.

### Statistical analysis

The data sets are tested for normality using the Jarque-Bera test. The normally distributed data sets are compared using the t-test whereas the Wilcoxon rank sum test is used for comparison of non-normal independent sets with a significance level p < 5%. The significant differences are reported wherever applicable. The applied method is presented in the text wherever applicable. The standard error of the mean (SEM) values are reported through the text and in the diagrams.

## Additional Information

**How to cite this article**: Abdi, E. *et al*. In a demanding task, three-handed manipulation is preferred to two-handed manipulation. *Sci. Rep*. **6**, 21758; doi: 10.1038/srep21758 (2016).

## Figures and Tables

**Table 1:** A summary of average objective measures of performance assessment over all the participants in the time spent in each task (2 h: two handed experiment, 3 h: three handed experiment).

		Total time for successful completion of the game (s)		Number of objects lost during the game	
Efficiency	*2* *h*	127 ± 7		3.8 ± 0.8	
	*3* *h*	126 ± 9		3.5 ± 0.8	
		**Left hand**	**Right hand**	**Foot**	
Workspace (cm^2^)	*2* *h*	Depends on the hand choice for caching the middle object.			
	*3* *h*	205 ± 25	179 ± 24	231 ± 26	
Travelled distance (cm)	*2* *h*	Depends on the hand choice for caching the middle object.			
	*3* *h*	442 ± 30	466 ± 25	555 ± 32	
Smoothness (η_sal)_	*2* *h*	−22 ± 2	−20 ± 1	—	
	*3* *h*	−21 ± 2	−22 ± 2	−29 ± 4	
Velocity (cm/s)	*2* *h*	4.8 ± 0.3	5.5 ± 0.5	—	
	*3* *h*	3 ± 0.9	2.9 ± 0.7	4.2 ± 0.9	
		**Left & Right hands**	**Left hand & Foot**	**Right hand & Foot**	**Left hand & Right hand & Foot**
Limbs’ simultaneous action time percentage	*2* *h*	33 ± 3%	—	—	—
	*3* *h*	13 ± 2%	6 ± 1%	4 ± 1%	5 ± 1%
		**Left hand**		**Right hand**	
Number of pinches	*2* *h*	62 ± 10		61 ± 11	
	*3* *h*	39 ± 6		42 ± 5	
Effort (J/kg)	*2* *h*	3.7 ± 0.6			
	*3* *h*	3.6 ± 0.7			

**Table 2:** Questionnaire statements for the three handed paradigm.

	Questionnaire statements
Q1	It felt natural for me to control three hands simultaneously.
Q2	It was easy for me to control the third hand by foot.
Q3	I got confused with the number of tasks that I had to perform simultaneously.
Q4	It was physically tiring for me.
Q5	It was mentally tiring for me.
Q6	I felt as if the virtual third hand was my own.
Q7	I felt as if my foot was turning into the third hand.
Q8	I felt as if my real hands were turning into the ‘virtual’ hands.

**Table 3:** Comparative questions for the two different paradigms.

	Questionnaire statements	The one with TWO hands	The one with THREE hands	NONE
Q1	In which experiment was it easier to catch the objects?			
Q2	Which strategy did you prefer for this game?			
Q3	Which experiment was mentally more tiring for you?			
Q4	Which experiment was physically more tiring for you?			

## References

[b1] Dong-Soo Kwon S.-Y. K. a. J. K. In Medical Robotics (ed Vanja Bozovic) Ch. 15, 197–218 (2008).

[b2] Kimura T., Umehara Y. & Matsumoto S. Laparoscopic cholecystectomy performed by a single surgeon using a visual field tracking camera - Early experience. Surgical Endoscopy-Ultrasound and Interventional Techniques 14, 825–829, doi: 10.1007/s004640000228 (2000). 11000362

[b3] Rane A., Kommu S., Eddy B., Rimington P. & Anderson C. Initial experience with the endoassist (R) camera holding robot in laparoscopic urological surgery. European Urology Supplements 6, 186–186, doi: 10.1016/s1569-9056(07)60650-2 (2007). PMC4247451 25484949

[b4] Finlay P. A. & Ornstein M. H. Controlling the movement of a surgical laparoscope - endosista(tm) with 4 degrees of freedom, operates in concert with surgeons intuitive head motions. IEEE Engineering in Medicine and Biology Magazine 14, 289–291, doi: 10.1109/51.391775 (1995).

[b5] Yavuz Y., Ystgaard B., Skogvoll E. & Marvik R. A comparative experimental study evaluating the performance of surgical robots aesop and endosista. Surgical laparoscopy, endoscopy & percutaneous techniques 10, 163–167, doi: 10.1097/00019509-200006000-00013 (2000). 10872979

[b6] Unger S. W., Unger H. M. & Bass R. T. AESOP robotic arm. Surgical Endoscopy 8, 1131 (1994). 7992194 10.1007/BF00705739

[b7] Niebuhr H. & Born O. Image Tracking System. A new technique for safe and cost-effective laparoscopic surgery. Chirurg 71, 580–584, doi: 10.1007/s001040050861 (2000). 10875019

[b8] Polet R. & Donnez J. Gynecologic laparoscopic surgery with a palm-controlled laparoscope holder. Journal of the American Association of Gynecologic Laparoscopists 11, 73–78, doi: 10.1016/s1074-3804(05)60015-1 (2004). 15104836

[b9] Schurr M. O. . Trocar and instrument positioning system TISKA - An assist device for endoscopic solo surgery. Surgical Endoscopy-Ultrasound and Interventional Techniques 13, 528–531, doi: 10.1007/s004649901029 (1999). 10227959

[b10] Jaspers J. E. N., Breedveld P., Herder J. L. & Grimbergen C. A. Camera and instrument holders and their clinical value in minimally invasive surgery. Surgical Laparoscopy Endoscopy & Percutaneous Techniques 14, 145–152, doi: 10.1097/01.sle.0000129395.42501.5d (2004). 15471021

[b11] Voorhorst F., Meijer D., Overbeeke C. & Smets G. Depth perception in laparoscopy through perception-action coupling. Minimally Invasive Therapy & Allied Technologies 7, 325–334, doi: 10.3109/13645709809152876 (1998).

[b12] Allaf M. E. . Laparoscopic visual field. Voice vs foot pedal interfaces for control of the AESOP robot. Surg Endosc 12, 1415–1418 (1998). 9822469 10.1007/s004649900871

[b13] Botvinick M. & Cohen J. Rubber hands ‘feel’ touch that eyes see. Nature 391, 756–756, doi: 10.1038/35784 (1998). 9486643

[b14] Riemer M. . The rubber hand illusion depends on a congruent mapping between real and artificial fingers. Acta Psychologica 152, 34–41, doi: 10.1016/j.actpsy.2014.07.012 (2014). 25103418

[b15] Miller L. E., Longo M. R. & Saygin A. P. Tool Morphology Constrains the Effects of Tool Use on Body Representations. Journal of Experimental Psychology-Human Perception and Performance 40, 2143–2153, doi: 10.1037/a0037777 (2014). 25151100

[b16] Bertamini M. & O’Sullivan N. The use of realistic and mechanical hands in the rubber hand illusion and the relationship to hemispheric differences. Consciousness and Cognition 27, 89–99, doi: 10.1016/j.concog.2014.04.010 (2014). 24842310

[b17] Longo M. R., Schuur F., Kammers M. P. M., Tsakiris M. & Haggard P. What is embodiment? A psychometric approach. Cognition 107, 978–998, doi: 10.1016/j.cognition.2007.12.004 (2008). 18262508

[b18] Newport R., Pearce R. & Preston C. Fake hands in action: embodiment and control of supernumerary limbs. Experimental Brain Research 204, 385–395, doi: 10.1007/s00221-009-2104-y (2010). 20012536 PMC2895889

[b19] Folegatti A., Farne A., Salemme R. & de Vignemont F. The Rubber Hand Illusion: Two’s a company, but three’s a crowd. Consciousness and Cognition 21, 799–812, doi: 10.1016/j.concog.2012.02.008 (2012). 22424939

[b20] Giummarra M. J., Gibson S. J., Georgiou-Karistianis N. & Bradshaw J. L. Mechanisms underlying embodiment, disembodiment and loss of embodiment. Neuroscience and Biobehavioral Reviews 32, 143–160, doi: 10.1016/j.neubiorev.2007.07.001 (2008). 17707508

[b21] Senguel A. . Force feedback facilitates multisensory integration during robotic tool use. Experimental Brain Research 227, 497–507, doi: 10.1007/s00221-013-3526-0 (2013). 23625046

[b22] Di Pino G., Maravita A., Zollo L., Guglielmelli E. & Di Lazzaro V. Augmentation-related brain plasticity. Frontiers in systems neuroscience 8, 109–109, doi: 10.3389/fnsys.2014.00109 (2014). 24966816 PMC4052974

[b23] Arezzo A. . Experimental trial on solo surgery for minimally invasive therapy - Comparison of different systems in a phantom model. Surgical Endoscopy-Ultrasound and Interventional Techniques 14, 955–959, doi: 10.1007/s004640000106 (2000). 11080411

[b24] Abdi E. B. M., Himidan S., Burdet E., Bleuler H. In New Trends in Medical and Service Robots Vol. 38 (ed H. Bleuler, Bouri, M., Mondada, F., Pisla, D., Rodić, A., Helmer, P.) 153–164 (Springer, 2015).

[b25] Llorens-Bonilla B., Asada H. H. & Asme. Control and coordination of supernumerary robotic limbs based on human motion detection and task petri net model. Asme 2013 Dynamic Systems and Control Conference, 2, doi: 10.1115/dscc2013-4083 (2013).

[b26] Wu F., Asada H. & Asme. SUPERNUMERARY ROBOTIC FINGERS: AN ALTERNATIVE UPPER-LIMB PROSTHESIS. (2014).

[b27] Balasubramanian S., Melendez-Calderon A. & Burdet E. A Robust and Sensitive Metric for Quantifying Movement Smoothness. IEEE Transactions on Biomedical Engineering 59, 2126–2136, doi: 10.1109/tbme.2011.2179545 (2012). 22180502

[b28] Moeller J. A word on standardization in longitudinal studies: don’t. Frontiers in Psychology 6, doi: 10.3389/fpsyg.2015.01389 (2015). PMC4569815 26441764

[b29] Abdi E., Bouri M., Himidan S., Burdet E. & Bleuler H. Third arm for surgeon: two hands versus three hands CARS 2015—Computer Assisted Radiology and Surgery Proceedings of the 29th International Congress and Exhibition Barcelona, Spain, June 24–27, 2015. International Journal of Computer Assisted Radiology and Surgery 10, 170–171, doi: 10.1007/s11548-015-1213-2 (2015). 25985879

[b30] Meesen R. L. J., Wenderoth N., Temprado J. J., Summers J. J. & Swinnen S. P. The coalition of constraints during coordination of the ipsilateral and heterolateral limbs. Experimental Brain Research 174, 367–375, doi: 10.1007/s00221-006-0471-1 (2006). 16819649

[b31] Nakagawa K., Muraoka T. & Kanosue K. Factors that determine directional constraint in ipsilateral hand-foot coordinated movements. Physiological reports 1, e00108–e00108, doi: 10.1002/phy2.108 (2013). 24303179 PMC3841043

[b32] Nakagawa K., Muraoka T. & Kanosue K. Potential explanation of limb combination performance differences for two-limb coordination tasks. Physiological reports 3, doi: 10.14814/phy2.12301 (2015). PMC4393209 25713327

[b33] McIsaac T. L. & Benjapalakorn B. Allocation of attention and dual-task effects on upper and lower limb task performance in healthy young adults. Experimental Brain Research 233, 2607–2617, doi: 10.1007/s00221-015-4333-6 (2015). 26080755

[b34] Simeone A. L., Velloso E., Alexander J. & Gellersen H. Feet Movement in Desktop 3D Interaction. 2014 Ieee Symposium on 3d User Interfaces (3dui), 71–74 (2014).

[b35] Abdi E., Burdet E., Bouri M. & Bleuler H. Control of a Supernumerary Robotic Hand by Foot: An Experimental Study in Virtual Reality. Plos One 10, doi: 10.1371/journal.pone.0134501 (2015). PMC4520700 26225938

